# A network pharmacology study on the *Tripteryguim wilfordii* Hook for treatment of Crohn’s disease

**DOI:** 10.1186/s12906-020-02885-9

**Published:** 2020-03-23

**Authors:** Jing Zhang, Qifeng Huang, Rui Zhao, Zhiyuan Ma

**Affiliations:** 1 Department of Pharmacy, Sir Run Run Shaw Hospital, School of Medicine, Zhejiang University, 3 East Qingchun Road, Hangzhou, 310016 China; 2 Department of Clinical Pharmacology, Key Laboratory of Clinical Cancer Pharmacology and Toxicology Research of Zhejiang Province, Affiliated Hangzhou First People’s Hospital, Zhejiang University School of Medicine, Hangzhou, Zhejiang 310006 People’s Republic of China

**Keywords:** *Tripterygium wilfordii* hook, Crohn’s disease, Network pharmacology, Anti-inflammatory agents

## Abstract

**Background:**

To explore the mechanism of action of *Tripterygium wilfordii* Hook (TWH) in the treatment of Crohn’s disease (CD) by network pharmacology.

**Methods:**

Traditional Chinese Medicine Systems Pharmacology database and analysis platform (TCMSP) was used to obtain the active constituents and targets of TWH. “Crohn’s disease” was used as a search term to search for related targets of CD from GeneCards database and OMIM database, thereby obtaining the targets of TWH against CD. The Cytoscape 3.7.1 software was used to construct a Chinese medicine compound-target network and STRING database to construct a protein-protein interaction network (PPI). The DAVID 6.8 online tool was used to perform gene ontology (GO) and kyoto encyclopedia of genes and genome (KEGG) pathway enrichment analysis of overlapping targets.

**Results:**

The database results showed that there were 30 active ingredients (14 key active ingredients) in TWH and 36 targets were screened out for CD treatment. Network analysis indicated that main targets of main active components of TWH were target genes such as VEGFA, MAPK8 and CASP3, which are involved in the regulation of cancer pathway, TNF signal pathway, hepatitis B pathway, apoptosis pathway, NF-kappa B signal pathway and so forth.

**Conclusions:**

TWH can play a multi-target and multi-channel synergistic treatment of CD by anti-angiogenesis, anti-apoptosis, anti-inflammation and immune regulation.

## Background

Crohn’s disease (CD) is a chronic inflammatory bowel disease with multi-factors that genetics and environment interact to manifest the disease [1]. It is typically characterized by transmural inflammation of the intestine and could affect any part of the gastrointestinal tract from mouth to anus. The entire layer of the intestinal wall can be affected, which can easily lead to complications such as intestinal obstruction, intestinal perforation, and an intestinal fistula. Meanwhile, abdominal pain, diarrhea, anemia, fever, and weight loss also consist of the common symptoms of active CD. The incidence of CD varies based on geographic region, ethnic groups and environment. For instance, CD’s prevalence is greater in developed regions than in developing areas. However, the number of people with CD has been steadily increasing, particularly in Eastern countries where some districts are undergoing fast urbanization [2]. Yet the cause of CD is still unknown, and the use of drugs currently cannot completely control or relieve the symptoms. It is urgent to develop more effective therapeutic strategies for affected patients. Recent studies have revealed that patients in North America and Europe using complementary and alternative medicines as a strategy has risen from 21 to 60%. And as an important part of complementary and alternative medicines, traditional Chinses medicine have been widely reported to treat inflammatory bowel disease [3].

*Tripterygium wilfordii* Hook (TWH) is a woody vine of the genus Tripterygium. It contains a variety of chemical components such as alkaloids, diterpenoids, triterpenoids and sesquiterpenes. As early as in Li Shizhen’s book “Compendium of Materia Medical” of the sixteenth century, there are records that TWH can anti-rheumatism, promote blood circulation, reduce swelling, and relieve pain. In recent years, clinical studies have found that TWH can effectively induce CD remission and maintain remission [4, 5], however, the specific mechanism of TWH in CD treatment is poorly understood. Network pharmacology is an approach to drug design that encompasses systems biology, network analysis, connectivity, redundancy and pleiotropy [6]. Compared with experimental pharmacology methods, network pharmacology emphasizes multi-channel regulation of signaling pathways, therefore especially suitable for the explanation of the mechanism of traditional Chinese medicine (TCM) with multiple chemical components and molecular targets.

**Fig. 1:**
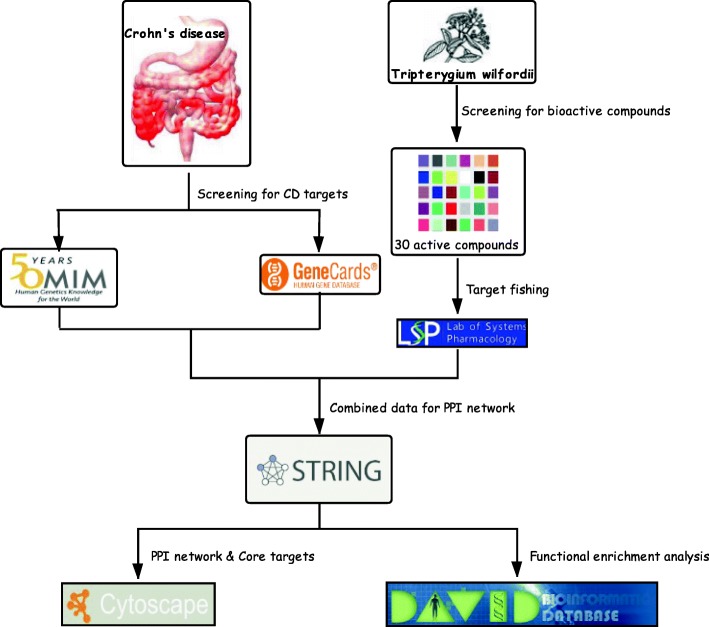
Detailed design and workflow of the present study

This study aimed to screen the bioactive components of TWH and elucidate the targets that contribute to its therapeutic effect, thereby further explaining its mechanism of action in CD treatment by network pharmacology. The study design and workflow are presented in Fig. 1.

## Methods

### Bioactive ingredient and target identification for *Tripterygium wilfordii* Hook (TWH)

The Chinese Medicine Systems Pharmacology Database and Analysis Platform (TCMSP) is a platform for integrating pharmacokinetics, medicinal chemistry, and drug-target-disease networks [7]. According to the TCMSP platform (http://lsp.nwu.edu.cn/tcmsp.php), the bioactive ingredients and targets of TWH were obtained. Bioactive ingredients (OB) refers to the rate and extent that a drug is absorbed into the body’s circulation. Drug-like properties (DL) reflects the nature of a drug which has a specific functional group or contains the same or similar physical characteristics. The human intestinal cell line Caco-2 is a useful tool for studying the absorption and transport of drugs in intestinal epithelial cells. The drug half-life reflects the concentration of the drug in the blood or body and is an important parameter for calculating the dosing interval, the dose administered, and the drug accumulation. Bioactive ingredients were collected based on the condition that OB > 30%, DL > 0.18, Caco-2 permeability > − 0.4, and half-life > 3 h as described previously [8]. Then the corresponding molecular targets of these collected active compounds were obtained by the same database.

### Target prediction of (TWH) in the treatment of Crohn’s disease (CD)

Search for CD related targets with “Crohn’s disease” as a search term using GeneCards database (https://www.genecards.org/) and the Online Mendelian Inheritance in Man (OMIM) database (http://omim.org/). The overlapping targets from CD treatment and from bioactive ingredients of TWH then allowed identification of targets of TWH in the treatment of CD.

### PPI network construction

The STRING database (https://string-db.org/) can be used to analyze the interaction between proteins and proteins [9]. In our study, the species was limited to “*Homo sapiens*”, the lowest interaction score was set to medium confidence (0.400), then discrete targets were hidden and the remaining parameters remained the default settings. Subsequently, the PPI network obtained in the STRING database was visualized and further analyzed using Cytoscape software.

### Core targets analysis

Cytoscape 3.7.1 is a visualization software that provides a more intuitive analysis of the mechanism of action of drugs in treating diseases. The node represents the chemical composition and potential target of the compounds in the TCM and the edge shows the relationship between TCM component and its target. The plug-in cytoHubba of the Cytoscape software was utilized to detect the significant targets from the PPI network [10]. The top 10 nodes were shown by the maximum neighborhood component algorithm.

### Enrichment analysis

The Database for Annotation, Visualization and Integrated Discovery (DAVID, https://david.ncifcrf.gov/) provides systematic, comprehensive biological annotation information for large-scale genes or proteins, and provides the most significantly enriched biological annotations [11]. Gene ontology (GO) and kyoto encyclopedia of genes and genome (KEGG) pathway enrichment of proteins in PPI network were carried out by DAVID, then the bubble plot of bioprocess and pathways were drawn by R software package.

## Results

### Bioactive ingredients collection

From TCMSP database, 30 bioactive components of TWH were obtained according to the OB, DL, Caco-2 and half-life values, as presented in Table 1.

**Table 1:** Information of 30 bioactive compounds in TWH

Molecular ID	Molecular name	OB (%)	Caco-2	DL	HL
MOL003182	(+)-Medioresinol di-O-beta-D-glucopyranoside_qt	60.69	0.45	0.62	3.05
MOL004443	Zhebeiresinol	58.72	0.53	0.19	3.32
MOL003279	99694-86-7	75.23	−0.13	0.66	3.71
MOL009386	3,3′-bis-(3,4-dihydro-4-hydroxy-6-methoxy)-2H-1-benzopyran	52.11	0.14	0.54	3.76
MOL003199	3,3′-bis-(3,4-dihydro-4-hydroxy-6-methoxy)-2H-2-benzopyran	61.85	0.02	0.54	3.97
MOL003189	WILFORLIDE A	35.66	0.31	0.72	4.05
MOL011169	Peroxyergosterol	44.39	0.86	0.82	4.06
MOL003187	triptolide	51.29	0.25	0.68	4.14
MOL003232	Triptofordin B1	39.55	0.41	0.84	4.19
MOL003211	Celaxanthin	47.37	1.73	0.58	4.33
MOL003196	Tryptophenolide	48.5	1.11	0.44	4.42
MOL003188	Tripchlorolide	78.72	0.16	0.72	4.44
MOL003192	Triptonide	67.66	0.15	0.7	4.48
MOL000211	Mairin	55.38	0.73	0.78	8.87
MOL003224	Tripdiotolnide	56.4	−0.29	0.67	4.91
MOL003198	5 alpha-Benzoyl-4 alpha-hydroxy-1 beta,8 alpha-dinicotinoyl-dihydro-agarofuran	35.26	−0.35	0.72	5.23
MOL000296	hederagenin	36.91	1.32	0.75	5.35
MOL000358	beta-sitosterol	36.91	1.32	0.75	5.36
MOL000449	Stigmasterol	43.83	1.44	0.76	5.57
MOL003184	81,827–74-9	45.42	0.85	0.53	5.58
MOL007415	[(2S)-2-[[(2S)-2-(benzoylamino)-3-phenylpropanoyl]amino]-3-phenylpropyl] acetate	58.02	0.32	0.52	6.03
MOL007535	(5S,8S,9S,10R,13R,14S,17R)-17-[(1R,4R)-4-ethyl-1,5-dimethylhexyl]-10,13-dimethyl-2,4,5,7,8,9,11,12,14,15,16,17-dodecahydro-1H-cyclopenta [a]phenanthrene-3,6-dione	33.12	0.9	0.79	6.56
MOL003266	21-Hydroxy-30-norhopan-22-one	34.11	0.9	0.77	6.66
MOL003235	Triptofordin D1	32	−0.35	0.75	7.2
MOL003244	Triptonide	68.45	0.15	0.68	4.79
MOL003209	Celallocinnine	83.47	0.89	0.59	10
MOL000422	kaempferol	41.88	0.26	0.24	14.74
MOL005828	nobiletin	61.67	1	0.52	16.2
MOL003280	TRIPTONOLIDE	49.51	0.72	0.49	17.94
MOL003217	Isoxanthohumol	56.81	0.76	0.39	17.98

### Target prediction of *Tripterygium wilfordii* Hook (TWH) in the treatment of Crohn’s disease (CD)

In our study, 62 targets of 14 bioactive components which had effect in the treatment of CD were obtained through TCMSP database, and 4206 genes were identified as the targets of CD from GeneCards and OMIM database in total. After mapping, 36 overlapping targets of WHT in the treatment of CD were collected.

### Network construction

**Fig. 2:**
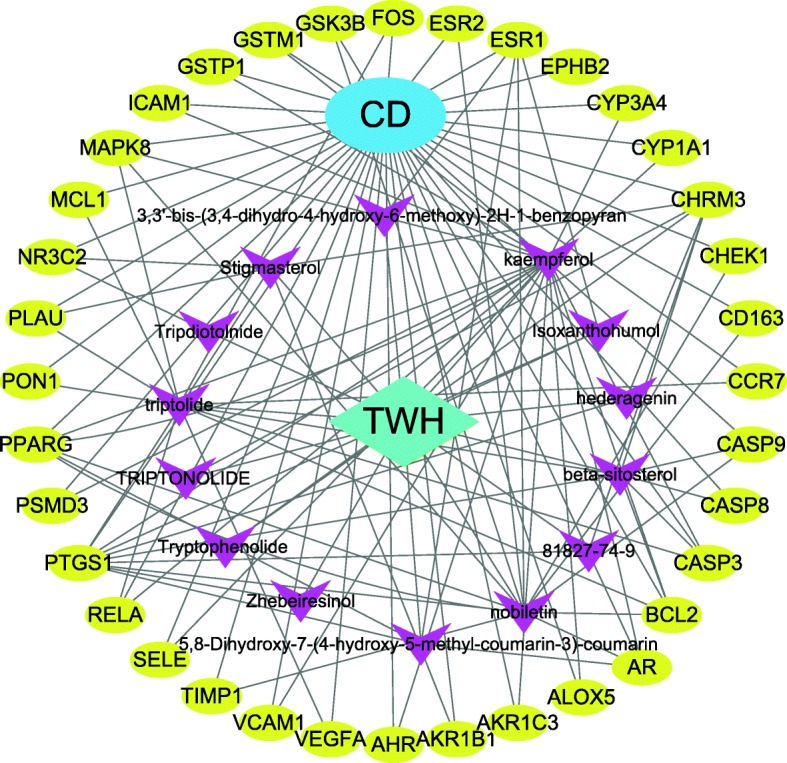
TWH-CD disease network. Purple inverted triangles represent active compounds of TWH, yellow circles are potential targets of TWH for the treatment of CD

**Fig. 3:**
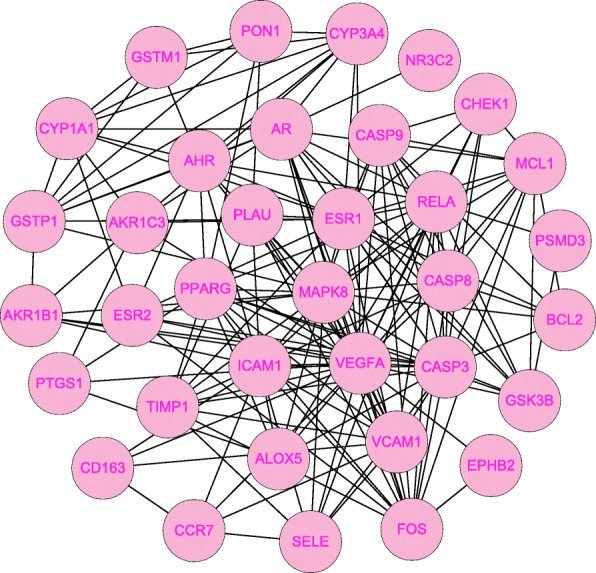
Network of overlapping targets

**Fig. 4:**
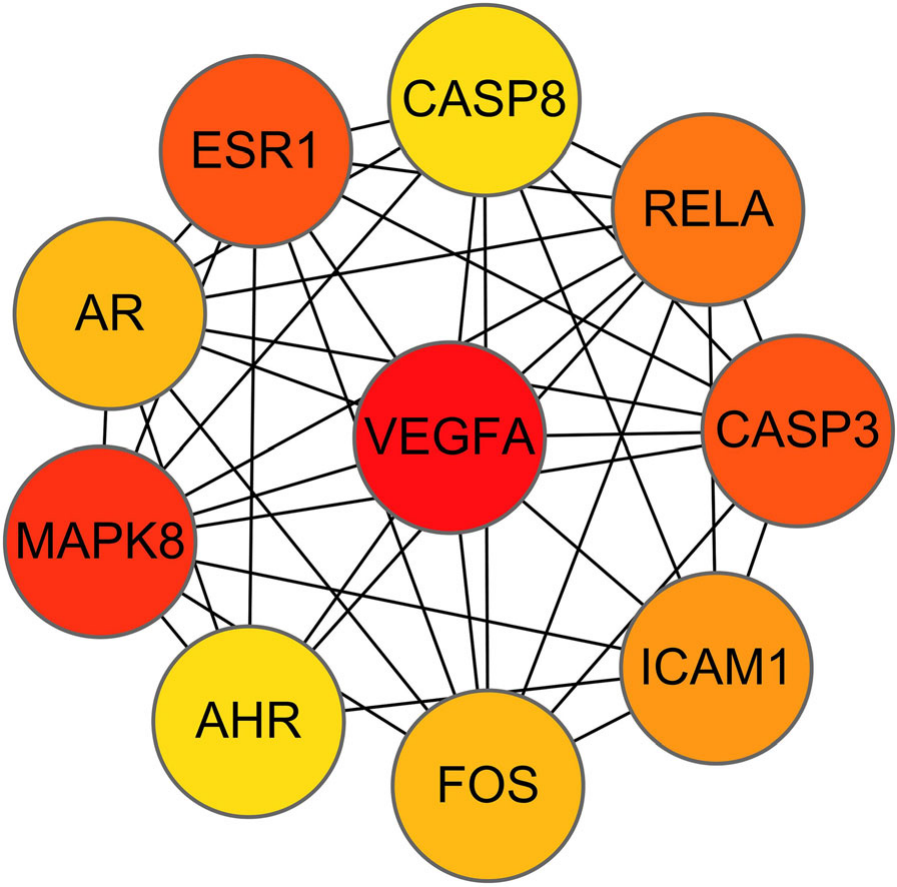
Hub genes of identified targets

The compound-target interaction network was constructed by Cytoscape 3.7.1 software. The network was found to have 52 nodes, including 14 bio-active molecules, 36 common target genes, 1 drug, 1 disease, and 118 edges, as shown in Fig. 2. PPI network reflects the spatiotemporal relationship of molecules within the cell and provides valuable information about molecular mechanisms in the physiological and pathological condition [12]. The 36 common targets were then inputted into the STRING database for PPI network analysis, then visualized by Cytoscape. There were 36 nodes and 178 edges in the PPI network, as shown in Fig. 3. The average node degree of freedom is 9.89, and the average aggregation coefficient is 0.666. The key targets were identified via cytoHubba plugin of Cytoscape. The top 10 targets were presented in Fig. 4. Besides, among all the core targets, the darker the red, the more important it was. It suggests that target genes such as VEGFA, MAPK8, CASP3 may play crucial roles in the treatment of CD.

### Go and KEGG pathway enrichment analysis

**Fig. 5:**
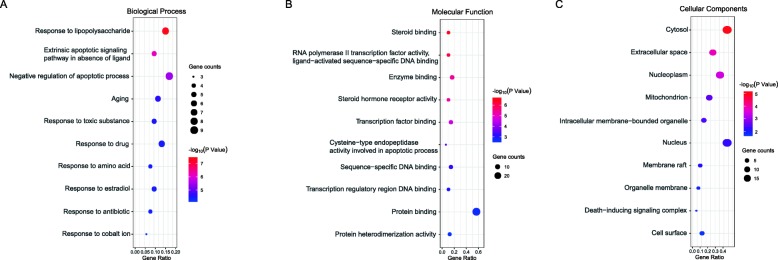
Gene Ontology analysis of potential targets of TWH. **a** Representative bubble plots of biological functional analysis of the core targets. **b** Representative bubble plots of molecular function among candidate targets. **c** Representative bubble plots of cellular components of identified targets. Gene ratio = count/set size

**Fig. 6:**
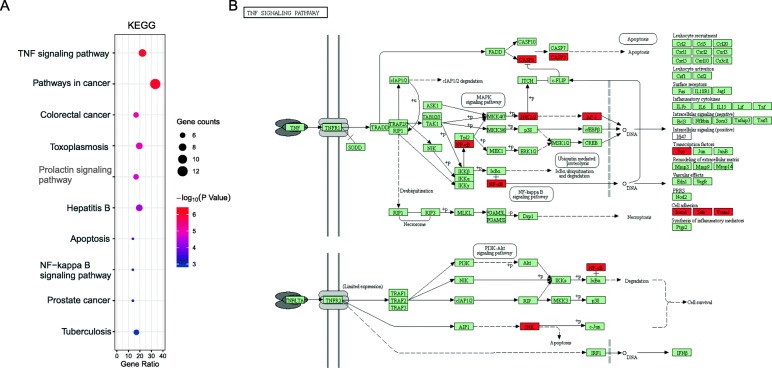
Signaling pathway enrichment analysis of core targets of TWH for CD. **a** Representative bubble plots of the pathway enrichment analysis of the core targets. Gene ratio = count/set size. **b** Reprinted of TNF signaling pathway with permission from Kyoto Encyclopedia of Genes and Genome

The results of DAVID database demonstrated that the target genes were mainly enriched in response to lipopolysaccharide, apoptosis, aging, drug reaction, reaction of toxic substances and so on in the biological processes (Fig. 5a); enzyme binding, transcription factor binding, RNA polymerase II core promoter proximal region sequence-specific binding, steroid binding and protein binding in molecular function (Fig. 5b); the cytosol, nucleoplasm and extracellular space in the cellular components (Fig. 5c). The pathway with the most enriched genes is the cancer pathway, with 12 genes; followed by the TNF signaling pathway, hepatitis B pathway, apoptotic pathway, NF-kappa B signaling pathway (Fig. 6a).

## Discussion

CD is an inflammatory bowel disease of unknown etiology and currently thought to have a relationship with infection, genetics, environment and cellular immunity. At present, the drugs for clinical treatment of CD are mainly divided into four categories, salicylic acid preparations, steroids, immunosuppressant preparation and biological agents. But there are still many problems we should reconsider, like allergy of salicylic acid, side effects of steroids, slow and short effect of immunosuppressant, expensive and easy recurrence of drug withdrawal of biological preparations. Nowadays, the TCM, tripterygium wilfordii glycosides derived from TWH have been widely used in immunoinflammatory diseases such as rheumatoid arthritis, lupus erythematosus, and atopic eczema [4, 13–15], are gradually being used for the treatment of CD.

Abnormal neovascularization in the intestinal tract, one of the typical pathological features of CD patients, is an important factor leading to chronic and persistent intestinal inflammations [16]. Vascular endothelial growth factor A (VEGFA) is the most vital pro-angiogenic factor, which can stimulate the secretion of various inflammatory cytokines and chemokines, thus accelerate the inflammatory process. In our study, we found that VEGFA can be affected by tripolide (MOL003187). Studies have shown that VEGF levels of patients are correlated with their CD clinical activity and appear to predict clinical response after anti-TNF-α treatment [17]. Some reported that VEGF levels decline faster and can maintain in low expression for a long time in those effectively treated patients [18]. In the Chinese population, people with variant genotype carriers of the VEGF gene rs3025039 locus had a lower risk of non-stenotic and non-penetrating CD. This may be related to the decrease of VEGF expression level, intestinal neovascularization and intestinal inflammatory reaction [19]. Our study also indicated that mitogen-activated protein kinase (MAPK) can be affected by kaempferol, nobiletin, and triptolide (MOL000422, MOL005828 and MOL003187). Continuous activation of extracellular regulated protein kinases, p38 kinase and c-Jun N-terminal kinase is observed in the inflammatory mucosa of CD patients [20], suggesting that MAPK transduction pathway is involved in the CD disease process. And we found that Capase-3 can be affected by kaempferol, beta-sitosterol, and triptolide (MOL000422, MOL000358 and MOL003187). Caspase-3 is an important gene that causes apoptosis and participates in the elevated apoptosis process of intestinal epithelial cells, which greatly impairs the epithelial barrier integrity [21]. Thus, previous and our study indicate VEGFA, MAPK8 and CASP3 may be potential targets of TWH in CD treatment, however it needs more vivo and invitro experiments evidence.

Tumor necrosis factor-α (TNF-α) is a member of a large family of proteins and receptors involved in immune regulation which interacts with two different receptors, and then initiates downstream signaling pathways to exert pro-inflammatory biological effects. In the inflammatory process of CD, TNF-α is an early pro-inflammatory cytokine and has been observed to be involved in the pathological processes in CD (including neutrophil accumulation, granuloma formation, increased epithelial permeability) [22]. Serum TNF-α is upregulated in patients with active CD activity compared with healthy control [23]. Triptolide, an active ingredient derived from TWH, could significantly reduce the production of TNF-α, IFN-γ and IL-4 in the colon [24]. In addition, it could ameliorate Th1-mediated chronic colitis and disordered immune state in IL-10(−/−) mice by inhibiting TNF-alpha/TNFR2 signal pathway [25]. NF-κB is a widely distributed nuclear transcription factor that regulates the expression of many inflammatory mediators including TNF-α, as presented in Fig. 6b. Abnormal activation of NF-κB starts at the initiation and progression stages of CD inflammation. Triptolide could inhibit the TLRs/NF-κB signaling pathway in vivo and in cultured colonic explants from CD patients [26].

However, due to the reported risk of toxic action on such organs as the liver, kidney, spleen, gastrointestinal tract or heart [27], application of TWH raises the question on the safety of its use in CD treatment. As toxicity was related to medication course, combined intervention, and drug dosage, the importance of follow-up animal experiments should be emphasized.

## Conclusions

In this paper, network pharmacology was used to explore the active components, potential targets and mechanisms of TWH in treating CD. The results showed that 14 ingredients of TWH may play important roles in biological processes of anti-angiogenesis, anti-apoptosis, anti-inflammatory and immune regulation by acting on VEGF, MAPK8, and CASP3 through TNF-α and NF-κB signaling pathways. More importantly, the potential targets and biological processes mentioned above may provide valuable information for further investigation into the mechanism of TWH for treating CD.

## Data Availability

The datasets used and analyzed during the current study are available from the corresponding author on reasonable request.

## Abbreviations

TWH: *Tripterygium wilfordii* Hook
CD: Crohn’s disease
TCMSP: Traditional Chinese Medicine Systems Pharmacology database and analysis platform
PPI: Protein-protein interaction network
GO: Gene ontology
KEEG: Kyoto encyclopedia of genes and genome
TCM: Traditional Chinese medicine
OMIM: Online Mendelian Inheritance in Man
DAVID: The Database for Annotation, Visualization and Integrated Discovery
VEGFA: Vascular endothelial growth factor A
MAPK: Mitogen-activated protein kinase
TNF-α: Tumor necrosis factor-α
